# Exploring the relationship between contextual factors and health‐promoting lifestyle profile (HPLP) among medical students: A cross‐sectional study

**DOI:** 10.1002/hsr2.2040

**Published:** 2024-04-21

**Authors:** Zahra Karimian, Mehrvash Moradi, Nahid Zarifsanaiey

**Affiliations:** ^1^ Department of E‐Learning in Medical Sciences, Virtual School and Center of Excellence in E‐Learning Shiraz University of Medical Sciences Shiraz Iran

**Keywords:** health, health‐promoting lifestyle profile (HPLP), lifestyle, medical student, nutrition, self‐actualization

## Abstract

**Background and Aims:**

The present study was conducted with the aim of investigating the relationship between health‐promoting lifestyle profile (HPLP) among medical students and contextual factors such as gender, age, field of study, academic level, marital status, history of physical and mental illnesses, and smoking.

**Methods:**

The present research was conducted in a cross‐sectional method in 2021 on 500 students of Shiraz University of Medical Students. An e‐questionnaire link was sent to them via email. The research tool was the HPLP questionnaire consisting of 52 questions in six domains in a 4‐point Likert scale. Data were analyzed using SPSS software version 24 with one‐sample *t*‐test, independent *t*‐test, analysis of variance, and Tukey's post hoc test.

**Results:**

A total of 500 students fully answered the questions completely. All components of a healthy lifestyle—except for exercise—obtained a score higher than the cut‐off point. The component of Self‐Actualization (spiritual growth) (3.035 ± 0.68) had the highest score, while the lowest score was related to exercise and physical activity (2.126 ± 0.60). Married individuals had a better average health score compared to singles (*p* = 0.047). The average health score did not have a significant relationship with gender, but significantly related to age, field of study (*p* < 0.001), history of mental illnesses (*p* < 0.001) and Smoking (*p* < 0.001).

**Conclusion:**

It seems that university officials should pay more attention to the health domain of students’ lifestyles, such as nutrition, the development of exercise and physical activities, and the management of stress and mental health. Additionally, providing necessary awareness and considering training courses, as well as developing sports and recreational facilities, can be effective in creating a better environment for the growth and development of students and ensuring their well‐being.

## 1 BACKGROUND

Lifestyle is one of the most important determinants of health, and most studies on health issues have shown that health behaviors significantly affect individuals’ health status.^1^ According to research, the quality of life is related to individuals’ lifestyles and behaviors.^2^ Moreover, it has been reported that more than half of the causes of mortality are related to people's lifestyles. Many health problems such as obesity, cardiovascular diseases, various types of cancer, and addiction, which are now prevalent in most countries, especially developing countries, are associated with changes in individuals’ lifestyles in that society.^3^

Currently, chronic diseases pose a serious threat to the health and longevity of people in countries worldwide. It was predicted that from 1990 to 2020, mortality caused by these diseases would increase by 77%, with the highest rates occurring in developing countries.^4^

Studies have shown that unhealthy behaviors have negative consequences on one's life, while healthy behaviors have positive effects on living a healthy and fulfilling life.^5^, ^6^

In this regard, students are a very important group who have a high degree of importance placed on their health and quality of life. This is because they play an important role in managing the future of society, and their health habits and behaviors will have a significant impact on their future quality of life.^7^ However, since students are often perceived to be in a relatively healthy stage of life due to their youth, they are less likely to pay attention to health‐promoting efforts around the world as a priority for them.^8^

Research has shown that unhealthy behaviors have become prevalent among students, who are an important segment of society. Wei^9^ and colleagues’ study in 2011 showed that Japanese students had low responsibility for their own health. Lee and Yuen Loke^10^ reported in 2005 that more than half of Chinese students skipped breakfast. The results of Dinevari and colleagues’ research in 2012 indicated poor performance of students regarding the consumption of healthy foods.^11^ Norouzinia^12^ and colleagues’ research in 2013 showed that students at Alborz University of Medical Sciences had a sedentary lifestyle.

In their study in 2014, Hosseinnejad and colleagues demonstrated that the lowest mean score was related to the exercise subscale, and the scores for health‐promoting behaviors and social and psychological health were significantly lower in students with a history of smoking or substance abuse.^7^ Moreover, some studies have shown that many students are potentially more susceptible to unhealthy lifestyles, such as smoking, unhealthy eating habits, increased stress, and sedentary lifestyles.^12^, ^13^ These evidences indicate that promoting and maintaining a healthy lifestyle, especially for students, is highly important and a significant challenge. The health‐promoting lifestyle model was the basis for the health behaviors of students in this study. According to 52‐item Health‐Promoting Lifestyle Profile (HPLP‐II) model developed by Walker^14^ and colleagues to determine the healthy lifestyle behaviors are comprised of six main components, including Self‐Actualization (spiritual growth), Health Responsibility, Exercise (physical activity), Nutrition, Interpersonal Support, and Stress Management.

In addition to assessing the health status of students, it is essential to examine the factors of their social and cultural status that influence their health‐promoting lifestyle.^15^ This includes aspects such as parental education, place of residence, occupation, and gender, which have been shown to impact students’ health behaviors. Some studies have shown that parental education level, place of residence, and occupation are related to the lifestyle of students.^16^ Gender is also a factor that has been shown to affect the dimensions of a healthy lifestyle in numerous studies.^6^, ^7^, ^17^, ^18^, ^19^, ^20^ This should be noted that studying at the university is a sensitive period of youth, especially because students are often independent and their lifestyle is less under parental supervision. Unhealthy behaviors that occur during this period may lead to increased health risks in later years.^17^ On the other hand, identifying health problems and lifestyles of students can help them better understand their health status and increase their knowledge and skills in improving healthy habits and behaviors.^21^, ^22^ A significant time of students is spent in the university environment. So university managers need to identify the health problems of students and plan for healthy nutrition, interpersonal support and communication skills development, and exercise.

Identifying these differences can help with better planning tailored to their needs. Therefore, the present study was conducted to determine the status of health‐promoting lifestyles and the factors affecting among medical students at one of the Iranian medical universities. The results of this study can be used to plan educational, developmental, and psychosocial programs to promote and encourage healthy behaviors among students. It should be noted that in the Iranian higher education system, medical universities are under the Ministry of Health, Treatment, and Medical Education, and are separate from universities where non‐medical students study, and therefore, non‐medical fields were not included in this study. Overall, the main objectives of this study were to examine the components of students’ health‐promoting lifestyle, and investigate its relationship with background variables, and assess the impact of physical and psychological status on it.

## 2 METHODS

### 2.1 Study design

The present study was conducted using a descriptive cross‐sectional method on the students of Shiraz University of Medical Sciences (SUMS) in 2021. SUMS is one of the largest universities in Iran with 17 main and satellite faculties and 53 research centers.

### 2.2 Sampling

The statistical population of this study consisted of all students enrolled at Shiraz in 2021, which was approximately 5000 participants. To determine the sample size, the Krejcie and Morgan table was used that it is based of Cochran formula.^23^ A similar study by Nilsaz et al.^24^ used the HPLP 52‐item questionnaire to determine the health status of medical students in Dezful, with approximately 35% of students being in good health and 65% being in fair or poor health. Considering the population size of 5000 students, a study confidence level of 95%, an estimated error rate of 0.05, a *Z*‐value of 1.96, and *p* and *q* values of 35% and 65%, respectively, the sample size was calculated using the following formula, and an acceptable sample size of approximately 327 individuals was obtained.

$$n=\frac{\frac{Z^{2}pq}{d^{2}}}{1+\frac{1}{N}\left(\frac{Z^{2}\;pq}{d^{2}}-1\right)}\frac{[1.96^{2}\times 0.35\times 065)/0.05^{2}}{1+[(1/5000)][(1.96^{2}\times 0.35\times 0.65)/0.05^{2}]-1}=327.$$

Given that many studies have reported low response rates for e‐questionnaires,^25^ we sent emails to 550 students and ultimately received 500 complete questionnaires. The sampling method was random sampling by lottery from the list of student emails.

### 2.3 Inclusion and exclusion criteria

The inclusion criteria were all students enrolled in Shiraz University of Medical Sciences in 2021 who participated in the study with consent, and the exclusion criteria were samples that had not answered more than 20% of the questions.

### 2.4 Data collection

After approval of the plan and obtaining ethical code from the ethics committee of Shiraz University of Medical Sciences and coordination with the university, the list of student emails was obtained with permission from the educational deputy. Then, an e‐questionnaire link was randomly emailed to 550 students.

### 2.5 Research instruments

The research tool included demographic information (age, gender, field of study, year of entry, etc.) and the 52‐item Health‐Promoting Lifestyle Profile questionnaire (HPLP‐Q) by Walker^14^ and colleagues. The HPLP‐Q consists of six components and 52 items, and its aim is to measure health‐promoting behaviors (Self‐Actualization [spiritual growth], Health Responsibility, Exercise [physical activity], Nutrition, Interpersonal Support, and Stress Management) with a 4‐point Likert scale (1 = never, 2 = sometimes, 3 = often, 4 = always). A total score of less than 50% indicates a poor lifestyle, 50%–75% indicates a moderate status, and 75%–100% indicates a good status and a healthy lifestyle. The validity and reliability of the HPLP tool have been confirmed in numerous studies.^7^, ^12^, ^14^

In this study, the Persian translated version of the 52‐item HPLP was used. The validity and reliability have been confirmed in previous research. In studiy of Mohammadi^26^ and college, the instrument was translated into Persian using Jones et al.^27^ approach and then back‐translated. A panel of experts examined the questionnaire for cultural sensitivities, clarity of the questions, differences, and errors in their meanings. The Persian version was evaluated, and its reliability (internal confidence) was 0.82 for the total scale and ranged from 0.64 to 0.91 for the subscales. Additionally, in the Mohammadi et al. study, a confirmatory factor analysis was conducted on the questionnaire, which yielded GFI and CFI values greater than 0.90, indicating a suitable fit.^26^ In another study, which used this tool on adolescents, the Content Validity Index of the HPLP‐II tool was calculated as 0.84 for the entire tool, and with exploratory and confirmatory factor analysis, it was confirmed with a good fit (RMSE = 0.066, NFI = 0.99, CFI = 0.99). The reliability of the tool was also confirmed with internal consistency of 0.86.^28^

To determine the cut‐off point, the value score of each option was divided by the number of options: (1 + 2 + 3 + 4) ÷ 4 = 2.5). In other words, a score higher than 2.5 indicates a suitable limit, and a score lower than that indicates an unsuitable status, and the minimum expected score is 50%.

### 2.6 Data analysis

To compare the overall mean and components of the questionnaire with the expected mean (cut‐off point), a one‐sample *t*‐test was used. To compare the means between two sub‐groups such as gender (Male/Female) and marital status (Single/Married), the independent *t*‐test with t index was used, and for comparisons between more than two sub‐groups such as age, field of study, academic level, and residency, the two‐way analysis of variance (ANOVA) with F index was used. Also, the correlation between the components of the questionnaires was examined using the Pearson correlation test. SPSS version 24 software was used for data analysis. The acceptable error rate was 5% and the confidence interval of the tests was 95%.

### 2.7 Ethics

All research activities were conducted in compliance with the relevant guidelines and regulations of the Research Vice‐Chancellor of Shiraz University of Medical Sciences (ID: 24129). The ethical aspects of the study were approved by the National Ethics Committee in Biomedical Research under the code IR.SUMS.REC.1400.680. In the introduction to the questionnaire, the research objectives were explained to the students. Written informed consent was obtained from all participants. The data was distributed, collected, compiled, and analyzed anonymously.

## 3 RESULTS

### 3.1 Demographic characteristics

A total of 500 questionnaires were completely filled out. Of this number, approximately 300 (60%) were female and 200 (40%) were male. In terms of age, 276 individuals (55.8%) were in the 18–25 age group, 147 (29.7%) in the 26–35 age group, and 72 (14.5%) were 36 years old and above. Regarding the field of study, 125 (25%) were in clinical medicine and dentistry, 85 (17%) in basic medical sciences (biochemistry, immunology, physiology, anatomy, etc.), 232 (46.5%) in paramedical fields (nursing, midwifery, healthcare, physiotherapy, etc.), and 58 (11.6%) were studying in other paramedical fields. A total of 181 (36.4%) were at the BSc level, 138 (27.8%) were pursuing a professional doctorate, 102 (20.5%) were at the MSc level, and 76 (15.3%) were pursuing a Ph.D. or were clinical residents. A total of 366 (73.3%) were single, and 134 (26.7%) were married. In terms of residency, 219 (43.8%) lived with their parents, 137 (27.4%) lived independently, and 144 (28.8%) resided in dormitories.

### 3.2 Status of health‐promoting lifestyle components

**Figure 1:**
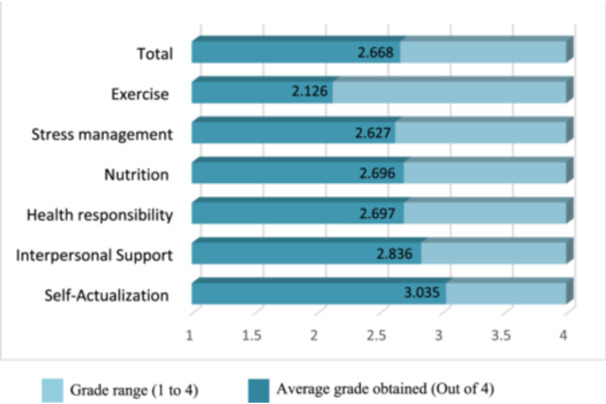
Mean score of HPLP components in medical students. HPLP, health‐promoting lifestyle profile.

To answer this question, the average ratings of the participants were examined using a one‐sample *t*‐test. Since the questions were in a 4‐point Likert scale, the cut‐off point or the expected minimum score for the current status and the importance level was considered to be 50% of the total score. The cut‐off point was 2.5. According to the findings, all components of the students’ lifestyle were relatively desirable or moderate. The highest mean score was related to the Self‐Actualization (spiritual growth) component (3.035 ± 0.68), and the lowest was related to exercise or physical activity (2.126 ± 0.60) (Figure 1).

### 3.3 Relationship between lifestyle and background variables

In response to the second question, the status of Health‐Promoting Lifestyle components was examined based on background variables. The relationship between the mean scores of the HPLP components and gender, and marital status was investigated using independent *t*‐tests. Additionally, the relationship between the HPLP components and age, place of residence, field of study, and educational level was investigated using ANOVA (Table 1).

**Table 1:** Total mean score of HPLP in medical students based on contextual variables.

Components		*N*	Mean	Std. D	*t*	*p*‐value
Gender	Female	293	2.68	0.479	1.17	0.239
	Male	197	2.63	0.505		
Marital status	Single	360	2.64	0.502	−1.99	0.047
	Married	131	2.74	0.452		

**Components**		***N***	**Mean**	**Std. D**	***F***	***p*‐value**
Age	18–25 Years	269	2.62	0.492	2.224	0.109
	26–35 Years	145	2.71	0.486		
	36–65 Years	71	2.71	0.475		
	Total	485	2.66	0.489		
Field of study	Clinical	123	2.59	0.461	6.379	<0.001
	Basic medical science	84	2.51	0.457		
	Para medical	227	2.75	0.501		
	None medical	57	2.72	0.491		
	Total	491	2.66	0.491		
Academic level	BSc	177	2.76	0.516	9.011	<0.001
	Professional doctorate	136	2.52	0.476		
	MSc	100	2.60	0.457		
	Ph.D./clinical residents	74	2.80	0.429		
	Total	487	2.66	0.492		
Place of residence	With parents:	212	2.66	0.515	0.500	0.607
	Independent	136	2.70	0.477		
	Dormitory	142	2.64	0.468		
	Total	490	2.66	0.491		

*Note*: Std. D: standard deviation; *N*: number; *t*: statistic index for Independent groups *t*‐test; *F*: statistic index for two‐way analysis of variance test (ANOVA).

Abbreviation: HPLP, health‐promoting lifestyle profile.

### 3.4 Gender

According to the results in Table 1, there was no significant difference in the overall mean score of health‐promoting lifestyle components by gender (*p* = 0.239). However, in the analysis of individual components, the results showed that in the components of health responsibility (*p* = 0.020), interpersonal support (*p* = 0.009), and nutrition (*p* = 0.007), females had a healthier lifestyle than males. However, in the exercise component, the mean score of males was higher than that of females (*p* = 0.001).

### 3.5 Marital status

The findings showed that the health‐promoting lifestyle of students is different based on their marital status (*p* = 0.047). In the analysis of individual components, the results showed that in the components of self‐actualization (spiritual growth) (*p* = 0.001), health responsibility (*p* = 0.006), interpersonal support (*p* = 0.012), nutrition (*p* = 0.017), and the total score (*p* = 0.047), married students had a better status compared to singles. Only in the exercise component, single individuals had a higher score (*p* = 0.021). However, there was no significant difference in stress management between the two groups (*p* = 0.521).

### 3.6 Age

The overall mean score of health did not show a significant difference by age groups (*p* = 0.109), but in the analysis of sub‐components, the components of self‐actualization (spiritual growth) (*p* = 0.009), health responsibility (*p* = 0.007), Interpersonal supprt (*p* = 0.048), and nutrition (*p* = 0.022) showed a significant difference by age groups, with higher mean scores in older age groups.

### 3.7 Field of study

According to the results, the overall mean score of health showed a significant difference by field of study (*p* < 0.001). In the analysis of sub‐components, the components of self‐actualization (spiritual growth) (*p* = 0.001), health responsibility (*p* = 0.001), nutrition (*p* = 0.001), and the total score (*p* = 0.001) showed significant differences by field of study. Additionally, the results of the Tukey test indicated that the mean score of clinical students was lower than other groups.

### 3.8 Academic level

The overall mean score of health had a significant difference by academic level (*p* < 0.001). In the analysis of sub‐components, the components of self‐actualization (spiritual growth) (*p* = 0.001), health responsibility (*p* = 0.001), stress management (*p* = 0.001), nutrition (*p* = 0.001), and the total score (*p* = 0.001) showed significant differences by academic level. The mean score of health in professional doctoral students was lower than other student groups, and the highest score was observed in students with postgraduate degrees.

### 3.9 Place of residency

Comparison of the health status components by place of residence showed no significant difference in the overall mean score (*p* = 0.607). However, in the components of health responsibility (*p* = 0.029) and nutrition (*p* = 0.030), the scores of dormitory residents were lower than other groups.

### 3.10 Medical and mental history

In response to the third research question, the extent to which the physical/medical and mental illnesses of students affect their healthy lifestyle was investigated. The results showed that a history of physical illness (Medical history) did not have a significant relationship with a healthy lifestyle (*p* = 0.148), but a significant relationship was found between mental history and a healthy lifestyle (*p* < 0.001). students without mental health problems had a better lifestyle than those with mental health disorders (Table 2).

**Table 2:** Total mean of HPLP components based on students’ medical and mental histories.

Components		*N*	Mean	Std. D	*t*	*p*‐value
Medical history	Yes	108	2.608	0.450	−1.447	0.148
	No	392	2.686	0.501		
Mental history	Yes	57	2.379	0.464	− 4.848	<0.001
	No	443	2.707	0.482		

*Note*: Std. D: standard deviation; *N*: number; *t*: statistic index for independent groups *t*‐test.

Abbreviation: HPLP, health‐promoting lifestyle profile.

### 3.11 Smoking

Although the percentage of students who smoked in the research sample was small, the comparison between the two groups showed that, in all cases, the average scores of healthy lifestyle components were lower in smokers than in those without a smoking history. This difference was significant in the components of spiritual growth (*p* = 0.002), health responsibility (*p* = 0.004), stress management (*p* < 0.001), nutrition (*p* < 0.001), and the overall score (*p* < 0.001) (Table 3).

**Table 3:** Comparison of health lifestyle components based on smoking status.

Components	Smoking history	*N*	Mean	Std. D	*t*	*p*‐value
Self‐actualization (spiritual growth)	Yes	51	2.7691	0.690	−3.165	0.002
	No	449	3.065	0.626		
Health responsibility	Yes	51	2.433	0.688	−2.870	0.004
	No	447	2.728	0.694		
Interpersonal support	Yes	51	2.703	0.576	−1.696	0.091
	No	447	2.852	0.593		
Stress management	Yes	51	2.291	0.526	−4.280	<0.001
	No	450	2.665	0.597		
Exercise (physical activity)	Yes	51	2.022	0.706	−1.033	0.302
	No	448	2.138	0.768		
Nutrition	Yes	51	2.413	0.581	−3.607	<0.001
	No	448	2.728	0.591		
Total	Yes	51	2.439	0.451	−3.574	<0.001
	No	440	2.695	0.489		

*Note*: Std. D: standard deviation; *N*: number; *t*: statistic index for Independent groups *t*‐test.

In examining the correlation between health components, the highest correlation was observed between stress management and nutrition (*r* = 0.580), Self‐Actualization (spiritual growth) and health responsibility (*r* = 0.564), nutrition and health responsibility (*r* = 0.563), Interpersonal Support and stress management (*r* = 0.561). Additionally, the highest correlation between the overall score and components were found in stress management (*p* = 0.798), health responsibility (*p* = 0.783), and nutrition (*p* = 0.781) (Table 4).

**Table 4:** Correlation between HPLP components of medical students.

Components correlation	1	2	3	4	5	6	Total
1. Self‐actualization	1						
2. Health responsibility	0.564^a^	1					
3. Interpersonal support	0.515^a^	0.540^a^	1				
4. Stress management	0.537^a^	0.514^a^	0.561^a^	1			
5. Physical activity	0.386^a^	0.390^a^	0.339^a^	0.513^a^	1		
6. Nutrition	0.519^a^	0.563^a^	0.467^a^	0.580^a^	0.479^a^	1	
7. Total	0.770^a^	0.783^a^	0.737^a^	0.798^a^	0.699^a^	0.781^a^	1

*Note*: *N* = 500.

Abbreviation: HPLP, health‐promoting lifestyle profile.

^a^ Correlation is significant at the 0.01 level (two‐tailed).

## 4 DISCUSSION

The results of this study showed that Self‐Actualization (spiritual growth) had the highest mean, while exercise (Physical activity) had the least. This finding is consistent with the study by Hosseinnejad et al.^7^ and Amiri et al.^15^ which was conducted on medical students in two university of Iran. Also Peker et al.^29^ conducted a study on dental students in Turkey,^19^ and Alzahrani et al.^6^ conducted a study on medical students in Saudi universities, both of which showed that the highest mean score was related to Self‐Actualization (spiritual growth). Perhaps this similarity is due to the common cultural and religious habits between these countries.

In the current study, the exercise and physical activity scores for students were the lowest, which align with the findings of Hosseinnejad et al.^7^ and Kirag et al.^30^ Both studies were conducted in medical universities. The reason for this may be that the participants in this study were medical students, who possess a higher level of health knowledge, but face a lack of time for exercise due to their engagement in academic and clinical training activities. Generally, the sports facilities of universities are provided when students are either engaged in indoor environments or in classrooms. In fact, students need to be provided with adequate time, resources and space for physical activity. It seems that adopting measures to enhance students’ access to sports facilities in dormitories or offering incentives for students to utilize nearby sports environments would be beneficial. Additionally, organizing sports competitions and events among students and valuing physical activities in personal and academic development is also encouraging.

Although studies have shown that increasing knowledge and awareness can affect health attitudes and behavioral habits,^31^ having more knowledge in the field of health does not necessarily imply correct health behaviors and habits. So, behaviors are affected by many variables such as participants’ sociodemographic variables, conditions, and facilities. This is supported by various studies.^6^, ^19^, ^31^, ^32^ Additionally, Shiraz University of Medical Sciences has scattered faculties and is not a comprehensive campus, so there are no centralized sports and cultural facilities for students, which can reduce their access to sports facilities. In other view, it can be concluded that although medical studies have shown that increasing knowledge and awareness can affect health attitudes and behavioral habits,^31^ having more knowledge in the field of health does not necessarily imply correct health behaviors and habits, as behaviors are affected by many variables such as people's sociodemographic variables, conditions, and facilities. This is supported by various studies.^6^, ^19^, ^32^, ^33^ On the other hand, in the present study, sports facilities at Shiraz University of Medical Sciences are only available to some faculties, and physical access to sports facilities is not equal for all faculties. This factor can be effective on this variable.

Another finding of the present research showed that there was a significant difference between the components of Self‐Actualization, health responsibility, Interpersonal Support, and nutrition by age group. Participants aged 36 and above had better scores compared to those aged 18–25. This finding is consistent with the results of the studies by Jalali Farahani et al.^34^ and Firoozabadi et al.^35^

In components of Self‐Actualization and spiritual grow, human development and moral theories indicates that individuals in older age groups tend to place a greater emphasis on ethics and human values. This is attributed to their wealth of experiences, which leads to deeper reflection on their past actions and a heightened need for spiritual matters.^36^

This score difference may also be related to the marital status of individuals, as in most cases, married individuals had higher scores in terms of health lifestyle. Perhaps this is because married students tend to consume healthier foods compared to singles. Younger age groups are more likely to have a tendency towards and consume unhealthy foods such as fast food, and snacks, while older students often take more caution in consuming risky foods or prioritize providing healthy food for their children. This is particularly important for women (mothers) in the family to ensure a healthy lifestyle, and women who play the role of homemaker have a significant role in providing healthy food.

This finding was also confirmed in the component of place of residence. The results showed that the components of health were related to the place of residence, although the total score was not significantly different by place of residence. However, in the components of health responsibility and nutrition, the scores of students living in dormitories were lower than other groups, which once again confirms the role of mothers and women in providing healthy life and food.

In general, it seems that since students spend a large part of their time in the university environment, their health is greatly affected by health planning at the university. Therefore, university administrators should pay attention to nutritional issues, provide sports facilities, create access to more facilities, and motivate students to observe correct nutritional behaviors. Additionally, regular monitoring of students’ health, including health tests, nutrition monitoring, and the development of sports environments as well as interfaculty and field competitions, can be effective.

According to the results of this study, there was a significant difference between the components of Self‐Actualization (spiritual growth), responsibility, support, exercise and physical activities, nutrition, and total score by marital status, with married individuals having higher averages in all cases. This finding is consistent with the results of the study by Mirzaei et al.,^37^ who stated that health‐promoting behaviors vary significantly by marital status and are more prevalent among married individuals than single individuals. Another point is that married individuals had better Interpersonal Support, but no significant difference was observed between the two groups in terms of stress management. Perhaps this is because a significant portion of individuals’ stress is due to financial and economic issues, which affects both single and married individuals.

Another finding of the study was that professional doctoral students received lower scores in most components of health lifestyle, while postgraduate students had better scores in most components. Although this may be partially due to age and marital status factors, one of the important reasons could be the high workload of professional doctoral students (such as medical and dental students) in clinical settings. Especially during their student years, a significant portion of their time is spent in hospital settings and work shifts, and due to their busy schedules and work in clinical environments, they have less time for health responsibility or nutrition and exercise. This finding is consistent with the results of the study by Danaei et al.,^38^ who reported that the average score of health promotion behaviors varied significantly by students’ gender and field of study.

Also, the results of the present study showed that there were significant differences in the components of responsibility, support, exercise, and nutrition by gender. Women had higher averages in health responsibility, Interpersonal support, and nutrition behaviors compared to men, while men had more physical activity and exercise on average. Overall, women had higher averages in health‐promoting behaviors compared to men. This finding is consistent with the results of the study by Rokhzadi et al.,^39^ who reported a significant relationship between gender and health responsibility for one's own health, exercise, and Interpersonal Support. It is also consistent with the results of studies by Alizadeh et al.,^40^ Mirzaei et al.,^37^ and Vary et al.,^41^ who reported similar findings.

Gender is one of the variables that can affect the level of physical activity and dietary habits, and awareness of these differences can be effective in health promotion programs. For example, in some countries, considering the cultural and social norms and the responsibilities that women have in the family, the lifestyle of men and women can be different; men have more free time to engage in sports activities, While women have to attend to household chores in addition to their work and academic activities. The results of the study by Oliveira^42^ and colleagues also showed that women mostly spend their time at home, while men are more interested in sports environments. Also women attach more importance to their nutrition compared to men. This result is consistent with similar research such as the results of the study by Wardle^43^ and colleagues on dietary behaviors in 23 countries showed that women reported higher consumption of high‐fat foods, fruit, and fiber, and limited salt intake compared to men. Women are more likely than men to diet and prioritize healthy eating. Gender differences in food choices are somewhat related to women's greater involvement in weight control and their stronger belief in healthy eating.

The present study showed that there was a significant difference only in the exercise components based on the history of physical/Medical illness. Individuals without a history of physical illness had a higher average of exercise activities than those with a history of physical illness. This finding was consistent with the results of Shafiei Alavijeh.^44^

It is natural that physical health has a significant impact on lifestyle and quality of life, especially in terms of influencing exercise and physical activity. However, another interesting point is that a history of physical illness did not have a significant impact on the healthy lifestyle of students, but a history of mental and psychological illness had a significant impact on almost all components, highlighting the importance of mental health and controlling these issues. In other words, students with physical illnesses or disabilities can have a healthy lifestyle similar to other students, but mental and psychological illnesses have a significant negative impact on lifestyle and overall health.^7^

The importance of mental and psychological issues in ensuring the health of students and their impact on all health components indicates that university administrators should identify mental and psychological disorders, provide counseling upon the arrival of students, and plan training related to anxiety control, interpersonal communication, and psychological counseling for students.

Finally, in examining the relationship between variables, healthy nutrition is correlated with most components. Perhaps the hidden point of this issue also confirms the impact of economic factors and the financial situation of households on providing healthy nutrition, as having healthy nutrition and consuming enough food is also influenced by financial factors. In fact, how students eat is not only influenced by their preferences, but also by their financial constraints in having a healthy meal. The nutrition index also somewhat indicates the students’ financial status. Furthermore, the highest correlation was observed between stress management components and total score, indicating the importance of psychological and spiritual aspects of life in having a healthy lifestyle. Additionally, high busyness and fast‐paced mechanical living reinforce an unhealthy lifestyle and nutrition. The results of research conducted at Japanese universities are also consistent with the present study, showing that Japanese students had low responsibility for their own health.^9^ Furthermore, the results of Li and Yuan Luoke's^10^ research in 2005 indicated that most Chinese students had eliminated breakfast from their diet.

### 4.1 Strengths and limitations

The present study's strengths include its large sample size from various fields of medical sciences at one of Iran's major universities and the use of a standard, validated instrument. However, a limitation is that the research sample came only from one medical university, so the results might be influenced by that institution's specific social and cultural characteristics.

## 5 CONCLUSION

Having a healthy lifestyle is an important factor in students’ health indicators in the future. A significant portion of students’ lives, which is the youthful and vibrant period in universities, is spent in an environment that is not conducive to good health due to the academic and occupational busyness, or neglecting the basic needs of a healthy lifestyle. In the present study, the role of nutrition, mental health, and stress management in having a healthy life was found to be crucial and had a higher average. It seems that university officials should pay more attention to the welfare of students because all three of these factors are among the essential life skills in today's world. Strengthening the stress management and mental health skills of students is of great importance. In this regard, the presence of knowledgeable counselors on mental health issues can be very helpful for students.

## 6 SUGGESTIONS FOR FUTURE STUDIES

Based on the findings of the present study, there are several suggestions for future research. First, it would be beneficial to investigate the impact of specific interventions, such as nutrition education programs or physical activity interventions, on the health behaviors of university students. Second, future studies could explore the role of cultural and social factors in shaping students’ health behaviors, particularly in the context of diverse student populations (Medical/None medical). Also, it would be valuable to examine the effectiveness of different approaches to promoting mental health and well‐being among university students, such as peer support programs or online counseling services. Finally, future research could investigate the long‐term impact of healthy behaviors on the academic performance and overall well‐being of university students.

## AUTHOR CONTRIBUTIONS

**Zahra Karimian**: conceptualization; data curation; investigation; methodology; project administration; resources; supervision; writing—original draft; writing—review & editing. **Mehrvash Moradi**: data curation; investigation. **Nahid Zarifsanaiey**: conceptualization; supervision.

## CONFLICT OF INTEREST STATEMENT

The authors declare no conflicts of interest.

## ETHICS STATEMENT

All research activities were conducted in compliance with the relevant guidelines and regulations of the Research Vice‐Chancellor of Shiraz University of Medical Sciences (ID: 24129). The ethical aspects of the study were approved by the National Ethics Committee in Biomedical Research under the code IR.SUMS.REC.1400.680. In the introduction to the questionnaire, the research objectives were explained to the students. Written informed consent was obtained from all participants. The data was distributed, collected, compiled, and analyzed anonymously.

## TRANSPARENCY STATEMENT

The lead author Zahra Karimian affirms that this manuscript is an honest, accurate, and transparent account of the study being reported; that no important aspects of the study have been omitted; and that any discrepancies from the study as planned (and, if relevant, registered) have been explained.

## Data Availability

The datasets used and/or analysed during the current study are available from the corresponding author on reasonable request. Corresponding author had full access to all of the data in this study and takes complete responsibility for the integrity of the data and the accuracy of the data analysis.
